# Clinical and laboratory characteristics of critical *Bordetella pertussis* in hospitalized infants in the Southwest region of Saudi Arabia

**DOI:** 10.3389/fped.2026.1881833

**Published:** 2026-07-20

**Authors:** Ali Alsuheel Asseri, Norah Alshehri, Ibrahim AlHelali, Ibrahim Al-Benhassan, Ahmed Alhijab Alhazmi, Khalid S. AlShehri, Abdullah Baoqbah, Ghada Haider, Syed Esam Mahmood, Ausaf Ahmad, Tarig Hamad

**Affiliations:** 1 Department of Child Health, College of Medicine, King Khalid University, Abha, Saudi Arabia; 2 Pediatric Intensive Care Units, Aseer Health Cluster, Abha, Saudi Arabia; 3 Department of Family and Community Medicine, College of Medicine, King Khalid University, Abha, Saudi Arabia; 4 Department of Community Medicine, Kalyan Singh Government Medical College Bulandshahr, Uttar Pradesh, India; 5 Department of Public Health, Aseer Health Cluster, Abha, Saudi Arabia

**Keywords:** clinical characteristics, critical pertussis, fulminant pertussis, infant death, laboratory findings, leukocytosis, pertussis, Saudi Arabia

## Abstract

**Background:**

Critical pertussis remains a critical health concern in infants and is characterized by severe leukocytosis, respiratory failure, and multisystem involvement. Despite global vaccination efforts, outbreaks continue to occur, particularly among unvaccinated or partially vaccinated infants. This study aimed to describe the clinical, laboratory, and management characteristics of critically ill infants with critical *Bordetella pertussis* in the southwest region of Saudi Arabia, with a focus on comparing survival and non-survival outcomes

**Materials and methods:**

A retrospective cohort study was conducted involving 70 infants hospitalized for severe pertussis between January 2024 and April 2025. Data collected included demographic details, presenting symptoms, comorbidities, vital signs, laboratory parameters [e.g., white blood cell (WBC) count, inflammatory markers, liver enzymes], treatments received (e.g., macrolides, exchange transfusions, leukoreduction therapies), and clinical outcomes. Statistical analyses included descriptive statistics and comparative tests (*t*-test, Mann–Whitney U test, chi-squared test).

**Results:**

Among the 70 infants, the median age was 3 months, with 46.4% of the infants aged < 3 months. Non-survivors (*n* = 4) exhibited significantly higher leukocyte counts (median WBC, 87.5 × 10^3^/µL) and neutrophil levels, profound hypoxemia, and higher inflammatory marker levels (C-reactive protein, erythrocyte sedimentation rate) than survivors. They also more frequently had coexisting conditions, such as congenital heart disease and genetic disorders. Clinical features, such as apnea, seizures, and hypoxemia, were strongly associated with death. All fatal cases involved severe pulmonary hypertension, which necessitated aggressive interventions, including exchange transfusions, inhaled nitric oxide, and inotropes. The laboratory findings of non-survivors showed marked leukocytosis, elevated liver enzyme levels, hyponatremia, and thrombocytopenia. Therapeutic efforts, including leukoreduction and advanced supportive measures, were more prevalent in non-survivors than in survivors but were insufficient to alter outcomes.

**Conclusion:**

Critical pertussis primarily affects unimmunized infants <3 months and is associated with severe illness and a notable fatality rate despite intensive care. Strengthening maternal and infant immunization, along with early risk identification and prompt intervention, is essential. Further research and public health efforts are needed to reduce disease burden.

## 1 Introduction

Despite widespread vaccination efforts, pertussis remains a significant cause of morbidity and mortality among infants worldwide (1, 2). Although global immunization programs have reduced the overall incidence, outbreaks continue, particularly among unvaccinated and partially vaccinated infants (3, 4). Regionally, pertussis continues to pose a substantial health burden, especially among infants aged < 6 months in the Eastern Mediterranean, with high hospitalization and complication rates (5). This underscores the ongoing need for effective prevention and management strategies, especially in vulnerable populations. Maternal vaccination with tetanus, diphtheria, and acellular pertussis (Tdap) has demonstrated effectiveness in decreasing early infant pertussis mortality, but coverage remains suboptimal owing to various barriers (6, 7). In Saudi Arabia, epidemiological data have revealed regional variations and periodic surges, highlighting the gaps in current prevention strategies (8, 9).

There is no universally accepted definition of critical or malignant pertussis; however, in observational studies, it is commonly described as a severe, rapidly progressive form of pertussis requiring intensive care admission, characterized by marked hyperleukocytosis (often ≥50 × 10⁹/L) alongside respiratory and/or circulatory failure necessitating intensive supportive care, and frequently associated with complications such as pneumonia, pulmonary hypertension, or shock (10–12).

Its pathophysiology involves pertussis toxin (PTx)–induced leukocyte proliferation, which increases blood viscosity and causes microvascular obstruction, contributing to pulmonary hypertension and right-sided heart failure (13, 14). Early identification of clinical predictors, such as young age, apnea, hypoxemia, and high leukocyte count, is crucial for prompt intervention (15, 16).

Management of malignant pertussis includes supportive care and leukoreduction therapies, such as exchange transfusion and leukapheresis. These interventions aim to reduce leukostasis, alleviate pulmonary hypertension, and improve survival outcomes (17). A case series suggested the potential benefits of early leukocyte reduction, but high-quality evidence remains limited, emphasizing the need for further studies (18, 19).

Given these various challenges, this study aimed to investigate the clinical presentation, laboratory findings, and management outcomes of critically ill infants with critical pertussis in southwestern Saudi Arabia. The rationale is to generate region-specific data that can inform early risk stratification; optimize clinical interventions, including leukoreduction techniques; and support public health initiatives, such as maternal immunization programs. Understanding disease patterns in this context is essential to reduce pertussis-related deaths and improve care for this vulnerable population.

## 2 Materials and methods

### 2.1 Study design and setting

We conducted a retrospective cohort study of infants hospitalized for critical pertussis in southwestern Saudi Arabia between January 2024 and April 2025. The patients were managed in a pediatric intensive care unit (PICU) at Abha Maternity and Children Hospital (AMCH). AMCH is a referral, tertiary care, and teaching hospital at Abha, the southwestern region's capital, Saudi Arabia.

### 2.2 Study participants and variable definitions

This retrospective cohort study included 70 infants hospitalized to PICU with confirmed pertussis between January 2024 and April 2025. Patients aged 1 month to 12 years with PCR-confirmed Bordetella pertussis and PICU stay >6 h were included; those diagnosed clinically or by serology alone were excluded. Screening for other respiratory pathogens were not performed.

Collected data included demographics, clinical features, comorbidities, vital signs, and laboratory parameters (CBC with differential, platelets, CRP, ESR, sodium, AST, ALT), as well as therapies (antibiotics, exchange transfusion, hydroxyurea, pulmonary vasodilators, corticosteroids). Outcomes included HFNC duration, mechanical ventilation, PICU/hospital length of stay, and survival/deaths.

Critical pertussis was defined as confirmed pertussis requiring admission to a pediatric intensive care unit. Malignant pertussis, which lacks a universally accepted definition, was defined in this study as a severe, rapidly progressive form of pertussis characterized by marked hyperleukocytosis (often ≥50 × 10⁹/L), accompanied by respiratory and/or circulatory failure requiring intensive care, and frequently associated with complications such as pneumonia, pulmonary hypertension, or shock (10–12).

### 2.3 Outcomes

The primary outcome was in-hospital death. Survivors and non-survivors were compared across the clinical, laboratory, and therapeutic domains.

### 2.4 Statistical analyses

Statistical analyses were performed using IBM SPSS Statistics (version 26.0). Continuous variables were assessed for normality using the Shapiro–Wilk test and are presented as means ± standard deviations for normally distributed data or medians with interquartile ranges (IQRs) for non-normally distributed data. Categorical variables are summarized as frequencies and percentages.

Survivors and non-survivors were compared using the independent samples *t*-test for normally distributed continuous variables and the Mann–Whitney U test for skewed data. The chi-squared or Fisher's exact test, as appropriate, was used to compare categorical variables. Statistical significance was set at a two-tailed *p*-value < 0.05.

## 3 Results

**Figure 1:**
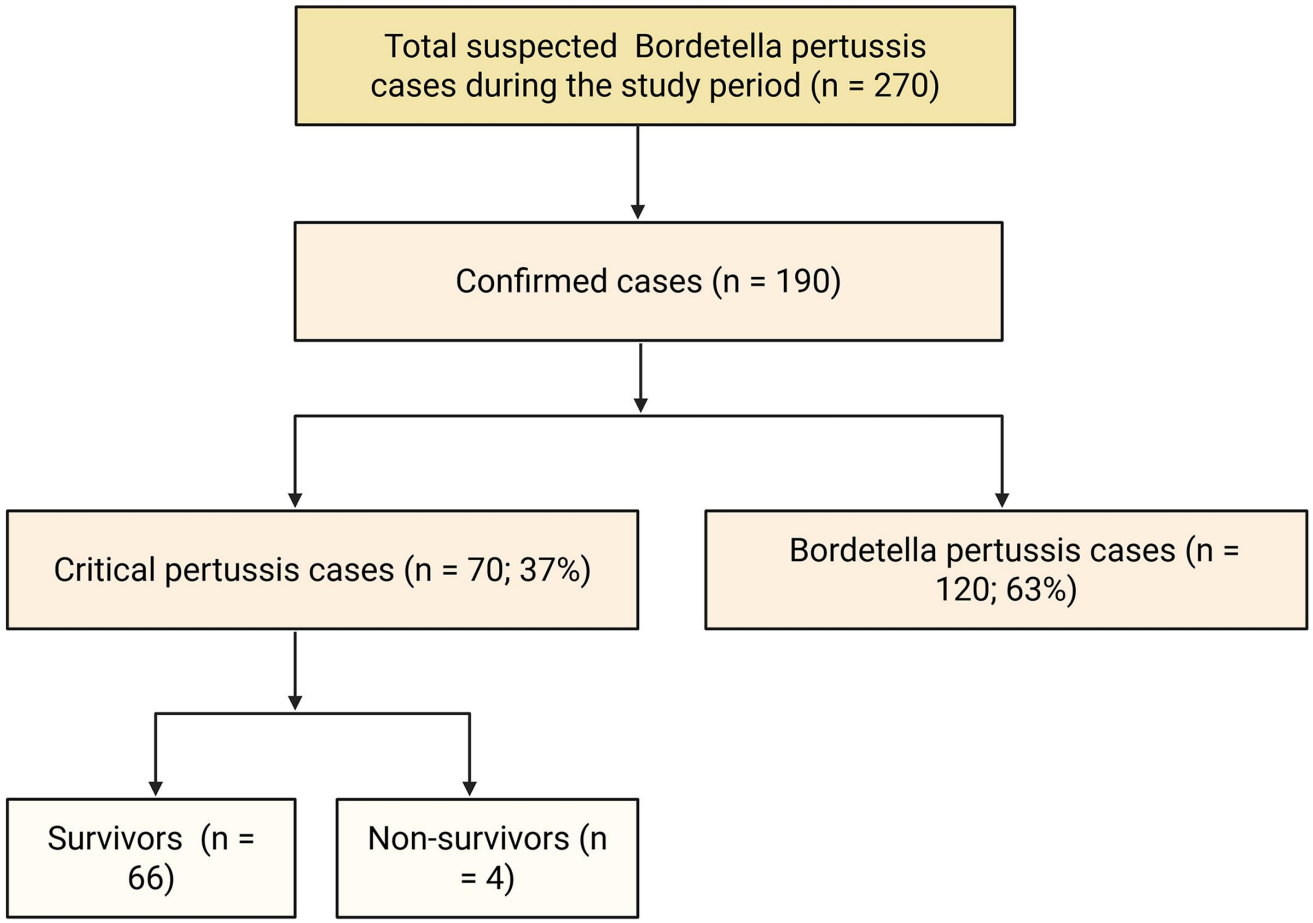
Flowchart of the enrolled patients.

Figure 1 illustrates the study cohort, with 190 confirmed pertussis cases, including 70 (37%) critical cases and 120 (63%) non-critical cases. Table 1 presents a demographic comparison of survivors and non-survivors hospitalized for critical pertussis, including symptoms, comorbidities, and vital signs.

**Table 1:** Characteristics of the enrolled patients stratified according to survival and death.

Variable	All cases (*n* = 70)	Survived (*n* = 66)	Died (*n* = 4)	*p*-value
Age, median (IQR), one missing	3.0 (1.0–4.0)	3.0 (1.0–4.5)	2.0 (1.0–3.0)	0.318
< 3 months, *n* (%)	32 (46.4)	29 (45.3)	2 (50.0)	0.814
3–12 months, *n* (%)	31 (44.9)	29 (45.3)	2 (50.0)	
> 12 months, *n* (%)	6 (8.7)	6 (9.4)	0 (0.0)	
Sex, male, *n* (%)	39 (55.7)	36 (54.5)	3 (75.0)	0.631
Premature (< 37 weeks GA), *n* (%)	7 (10.1%)	7 (10.9)	0 (0.0)	–
Hospital stay, days, median (IQR)	9.0 (5.0–15.0)	8.5 (5.0–15.0)	9.0 (2.75–22.75)	0.783
Symptoms, *n* (%)				
Paroxysmal spasmodic cough	57 (83.8)	52 (82.5)	4 (100.0)	–
Inspiratory whoop	14 (20.3)	16 (21.9)	0 (0.0)	0.574
Nasal congestion/rhinorrhea	26 (39.4)	25 (41.0)	0 (0.0)	0.154
Paroxysmal cyanosis	32 (46.4)	32 (50.0)	0 (0.0)	0.117
Facial flushing and/or cyanosis during coughing	13 (18.8)	28 (18.8)	0 (0.0)	–
Post-tussive vomiting	14 (20.6)	14 (22.2%)	0 (0.0)	0.572
Apnea	36 (52.2)	32 (48.5)	4 (100.0)	< 0.001
Hypoxemia	23 (32.9)	19 (28.8)	4 (100.0)	0.002
Seizure	8 (11.4)	6 (9.1)	2 (50.0)	0.03
Fever	22 (31.4)	21 (31.8)	1 (25.0)	0.99
Coexisting medical conditions, any, *n* (%)	25 (35.7)	21 (31.8)	4 (100.0)	0.003
Congenital heart disease	9 (12.9)	6 (9.1)	3 (75.0)	< 0.001
Genetic conditions	5 (7.1)	3 (4.5)	2 (50.0)	0.01
Hydronephrosis	4 (5.7)	4 (6.1)	0 (0.0)	0.99
Laryngomalacia	6 (8.6)	5 (7.6)	1 (25.0)	0.28
Neuroblastoma	2 (2.9)	1 (1.5)	1 (25.0)	0.06
Seizure disorder (preexisting)	5 (7.1)	3 (4.5)	2 (50.0)	0.01
Vital signs at admission				
RR (/min), mean ± SD	48 ± 12	46 ± 10	62 ± 15	0.02
HR (/min), mean ± SD	158 ± 18	155 ± 15	178 ± 22	0.01
Oxygen saturation (%), mean ± SD	89 ± 4	91 ± 3	82 ± 5	< 0.001
Body temperature ( °C), mean ± SD	38.1 ± 0.7	38.0 ± 0.6	38.9 ± 0.8	0.04

IQR, interquartile range; GA, gestational age; RR, respiratory rate; SD, standard deviation; HR, heart rate.

Among the 70 pediatric cases included in this study, the median age was 3.0 (IQR, 1.0–4.0) months, with no significant difference between survivors and non-survivors (*p* = 0.318). Nearly half of the cohort (46.4%) was younger than 3 months. Males accounted for 55.7% of the population, and premature birth was documented in 10.1% of the patients; however, none of the deceased patients were premature. The median duration of hospitalization was 9 (IQR, 5.0–15.0) days and was similar across outcome groups (*p* = 0.783).

Paroxysmal spasmodic cough was the most prevalent symptom (83.8%), followed by apnea (52.2%), cyanotic episodes (46.4%), and nasal congestion or rhinorrhea (39.4%). Several symptoms demonstrated strong associations with death, being significantly more common among non-survivors than among survivors, including apnea (100% in non-survivors vs. 48.5% in survivors; *p* < 0.001), hypoxemia (100% vs. 28.8%, *p* = 0.002), and seizures (50.0% vs. 9.1%, *p* = 0.03).

Coexisting medical conditions were present in 35.7% of the cohort and were significantly associated with death (*p* = 0.003). In particular, CHD showed a strong association with fatal outcomes (75.0% among non-survivors vs. 9.1% among survivors, *p* < 0.001). Genetic disorders (*p* = 0.01) and preexisting seizure disorders (*p* = 0.01) were also markedly more frequent among the children who died.

At admission, non-survivors demonstrated significantly more severe physiological derangements, having higher respiratory rates (62 ± 15 vs. 46 ± 10 breaths/min, *p* = 0.02), higher heart rates (178 ± 22 vs. 155 ± 15 beats/min, *p* = 0.01), and substantially lower oxygen saturation levels (82% ± 5 vs. 91% ± 3, *p* < 0.001) than survivors. Body temperature was slightly but significantly higher in non-survivors than in survivors (38.9 °C ± 0.8 vs. 38.0 °C ± 0.6, *p* = 0.04).

Overall, death in this cohort was associated with specific symptoms (apnea, hypoxemia, seizures), the presence of significant comorbidities—particularly CHD—and pronounced abnormalities in vital signs at presentation.

Laboratory evaluations demonstrated marked differences between survivors and non-survivors, with several parameters showing strong associations with death Table 2. The overall cohort exhibited substantial leukocytosis, with a median white blood cell (WBC) count of 31 × 10^9^/L (IQR, 16–58); however, WBC levels were more profoundly elevated among non-survivors than among survivors, reaching a median of 87.5 × 10^9^/L (IQR, 56.25–118.25) as opposed to 27.0 × 10^9^/L (IQR, 16.0–56.0) in survivors (*p* = 0.0122). A similar pattern was observed in absolute neutrophil count, with markedly greater elevation in non-survivors (88 × 10^9^/L; IQR, 61–202) than in survivors (8.1 × 10^9^/L; IQR, 5.2–11.4; *p* < 0.001), indicative of severe neutrophilic leukocytosis among fatal cases.

**Table 2:** Comparison of laboratory characteristics of survivors and non-survivors.

Laboratory and imaging results	Reference range	All cases (*n* = 70), median (IQR)	Survived (*n* = 66), median (IQR)	Died (*n* = 4), median (IQR)	*p*-value
WBC ( × 10^3^/µL)	4.3–11.0	31 (16–58)	27.0 (16.0–56.0)	87.5 (56.25–118.25)	0.012
Hyperleukocytosis (WBC ≥ 50 × 10^9^/L), *n* (%)	-	22 (31.4)	19 (28.7)	3 (75.0%)	0.096
ANC ( × 10^9^/L)	1.5–8.5	8.6 (5.4–12.9)	8.1 (5.2–11.4)	88 (61–202)	< 0.001
AEC ( × 10^9^/L)	0.05–0.7	0.18 (0.09–0.32)	0.21 (0.11–0.35)	0.02 (0.00–0.04)	0.028
ALC ( × 10^9^/L)	2.0–11.0	16.1 (10.24–32)	14.63 (10.0–27.26)	67.5 (43.75–95.52)	0.003
Platelets ( × 10^9^/L)	150–400	312 (246–402)	328 (265–415)	137 (112–171)	0.021
CRP (mg/dL)	0.0–0.9	2 (1–11.5)	1.7 (0.3–6)	20.5 (16.75–25.25)	0.016
ESR (mm/hr)	0–15	6 (5–21)	6 (5–18)	37 (24.75–50)	0.018
Sodium (mEq/L)	135–145	134 (131–137)	135 (132–138)	128 (122–129)	0.017
AST (U/L)	10–50	48 (32–71)	45 (30–62)	137 (96–171)	0.006
ALT (U/L)	10–40	39 (28–56)	36 (26–49)	65 (41–82)	0.008

WBC, white blood cell (count); ANC, absolute neutrophil count; AEC, absolute eosinophil count; ALC, absolute lymphocyte count; CRP, C-reactive protein; ESR, erythrocyte sedimentation rate; Sodium, serum sodium; AST, aspartate aminotransferase; ALT, alanine aminotransferase.

When patients were stratified according to the presence of hyperleukocytosis (WBC > 50 × 10⁹/L), this finding was identified in 19 of 66 survivors (28.7%) and 3 of 4 non-survivors (75.0%). Although hyperleukocytosis was more common among non-survivors, the difference did not reach statistical significance (Fisher's exact test, *p* = 0.096).

The absolute lymphocyte count (ALC) also significantly differed between the groups. Although the median ALC for the entire cohort was 16.1 × 10^9^/L (IQR, 10.24–32), non-survivors again had markedly higher values (67.5 × 10^9^/L; IQR, 43.75–95.52) compared with survivors (14.63 × 10^9^/L; IQR, 10.0–27.26; *p* = 0.00335). In contrast, absolute eosinophil count was significantly lower among non-survivors (0.02 × 10^9^/L; IQR, 0.00–0.04) than in survivors (0.21 × 10^9^/L; IQR, 0.11–0.35; *p* = 0.028).

Thrombocytopenia was another prominent finding among fatal cases. Although platelet counts were generally within the reference range for the full cohort (median, 312 × 10^9^/L; IQR, 246–402), non-survivors had substantially reduced counts (137 × 10^9^/L; IQR, 112–171), significantly lower than those of survivors (328 × 10^9^/L; IQR, 265–415; *p* = 0.021).

Markers of inflammation were markedly elevated in non-survivors. CRP level reached a median of 20.5 mg/dL (IQR, 16.75–25.25) among fatal cases, markedly greater than the 1.7 mg/dL (IQR, 0.3–6) in survivors (*p* = 0.0157). The ESR was also significantly higher in non-survivors (37 mm/h; IQR, 24.75–50) than in survivors (6 mm/h; IQR, 5–18; *p* = 0.0183).

Electrolyte abnormalities were also evident, with non-survivors demonstrating more pronounced hyponatremia (median sodium, 128 mEq/L; IQR, 122–129) compared with survivors (135 mEq/L; IQR, 132–138; *p* = 0.017).

Liver enzyme elevation was more prominent among non-survivors than among survivors. AST levels were significantly higher in the fatal cases (137 U/L; IQR, 96–171 in the survivors (45 U/L; IQR, 30–62; *p* = 0.006). The same was true for ALT, with elevated values in non-survivors (65 U/L; IQR, 41–82) relative to survivors (36 U/L; IQR, 26–49; *p* = 0.008).

Overall, fatal outcomes were characterized by extreme leukocytosis (predominantly neutrophilic), markedly elevated inflammatory markers, thrombocytopenia, hyponatremia, and significant hepatic enzyme elevation, collectively indicating a severe systemic inflammatory response and multiorgan involvement.

Table 3 shows the comparison of the therapeutic approaches and clinical outcomes between survivors and non-survivors. Macrolide therapy was the mainstay of treatment in both groups and was administered to 92.4% of the survivors and 75.0% of the non-survivors. However, the difference was not statistically significant (*p* = 0.24). Broad-spectrum antibiotic use was similarly high in both groups (93.9% vs. 100%, *p* = 1.00), reflecting routine empirical coverage in severe pediatric respiratory infections.

**Table 3:** Comparison of therapies, duration of supportive care, and outcome measures among the survivor and non-survivor groups.

Variable	Survived (*n* = 66)	Died (*n* = 4)	*p*-value
Macrolides therapy, *n* (%) (*n* = 64)	61 (92.4%)	4 (100%)	1.00
^a^Broad-spectrum antibiotics, *n* (%) (*n* = 66)	62 (93.9%)	4 (100%)	1.00
Exchange transfusion, *n* (%) (*n* = 14)	10 (15.2%)	4 (100%)	0.001
Hydroxyurea, *n* (%) (*n* = 5)	3 (4.5%)	2 (50.0%)	0.01
Inhaled nitric oxide and/or sildenafil, *n* (%) (*n* = 4)	0 (0%)	4 (100%)	< 0.001
Corticosteroids, *n* (%) (*n* = 18)	14 (21.2%)	4 (100%)	0.002
Length of HFNC use (days), median (IQR)	3 (2–5)	6 (4–9)	0.04
Length of mechanical ventilation (days), median (IQR)	6 (4–12)	3.5 (1.5–25.5)	0.610
Length of PICU stay (days), median (IQR)	4 (2–7)	9 (2.5–30.5)	0.404
Length of hospital stay (days), median (IQR)	9 (5–15)	9 (2.5–30.5)	0.88
Duration of oxygen therapy (days), median (IQR)	4 (2–7)	12 (8–20)	0.01

HFNC, high-flow nasal cannula; IQR, interquartile range; PICU, pediatric intensive care unit.

a Broad-spectrum antibiotics utilized included ceftriaxone, meropenem, vancomycin, and/or piperacillin/tazobactam.

In contrast, several advanced or rescue therapies were used with a significantly greater frequency among non-survivors than among survivors. Exchange transfusion was required in all fatal cases (100%), compared with only 15.2% of survivors (*p* = 0.001), underscoring its association with severe disease physiology. The use of hydroxyurea also markedly differed, being administered to 50.0% of non-survivors compared with 4.5% of survivors (*p* = 0.01). Additionally, all non-survivors received inhaled nitric oxide and/or sildenafil, a therapy not required by any survivor (*p* < 0.001), indicating the presence of severe pulmonary hypertension among fatal cases. Corticosteroid administration was also significantly more common in non-survivors than in survivors (100% vs. 21.2%, *p* = 0.002), suggesting its use as escalation therapy in critically ill children.

Supportive care duration distinguished the outcome groups similarly. Non-survivors required a significantly longer period of HFNC support than survivors, with a median duration of 6 (IQR, 4–9) days vs. only 3 (IQR, 2–5) days in survivors (*p* = 0.04). Mechanical ventilation duration did not differ significantly between groups, with survivors having a median duration of 6 days (IQR, 4–12) compared to 3.5 days (IQR, 1.5–25.5) among non-survivors (*p* = 0.610). Similarly, the length of PICU stay showed no statistically significant difference, with survivors having a median stay of 4 days (IQR, 2–7) and non-survivors 9 days (IQR, 2.5–30.5; *p* = 0.404).

Despite these differences in intensive care metrics, the total length of hospital stay did not significantly differ between the groups (*p* = 0.88), likely reflecting the variability within the small number of fatal cases. Nevertheless, the duration of oxygen therapy was substantially longer among non-survivors than survivors, at a median of 12 (IQR, 8–20) days, vs. just 4 (IQR, 2–7) days in survivors (*p* = 0.01), consistent with greater respiratory compromise among non-survivors.

Overall, the need for high-intensity therapies, including exchange transfusion, advanced pulmonary hypertension management, corticosteroids, and prolonged ventilatory support, was strongly associated with death. These findings highlight the trajectory of rapid escalation and refractory disease observed in the most severely affected patients.

Detailed of the four deceased cases are summarized in Table 4. All four fatal cases occurred in young, unimmunized infants between 1 and 3 months of age, consisting of three males and one female. None of the children had received a pertussis vaccination prior to the onset of illness. Each patient presented with severe pneumonia, high fever, and rapidly progressive respiratory distress culminating in respiratory failure. Cardiac failure was also documented in all four infants, reflecting the multisystem involvement characteristic of fulminant pertussis.

**Table 4:** Clinical characteristics of the 4 non-survivor cases.

Clinical feature	Case 1	Case 2	Case 3	Case 4
Age (months)/sex	1/M	3/F	1/M	3/M
Pertussis vaccination	No	No	No	No
Severe pneumonia	Yes	Yes	Yes	Yes
Fever	Yes	Yes	Yes	Yes
Peak WBC ( × 10^9^/L)	60	128	45	115
Peak ALC ( × 10^9^/L)	45	111	40	90
Peak CRP (mg/dL)	10	22	35	19
Peak ESR (mm/hr)	12	45	29	65
Length of hospitalization (days)	60	45	46	15
Respiratory failure	Yes	Yes	Yes	Yes
Cardiac failure	Yes	Yes	Yes	Yes
Severe pertussis	Yes	Yes	Yes	Yes
Inhaled nitric oxide/sildenafil	Yes	Yes	Yes	Yes
Vasoactive support needed	Yes	Yes	Yes	Yes
Cause of death	Respiratory + cardiac failure	Respiratory + cardiac failure	Respiratory + cardiac failure	Respiratory + cardiac failure

WBC, white blood cell (count); ANC, absolute neutrophil count; AEC, absolute eosinophil count; ALC, absolute lymphocyte count; CRP, C-reactive protein; ESR, erythrocyte sedimentation rate.

Marked leukocytosis was a consistent feature among the fatal cases, with peak WBC counts ranging from 45 × 10^9^/L to 128 × 10^9^/L. Lymphocytosis was similarly profound, with ALCs between 40 × 10^9^/L and 111 × 10^9^/L. Inflammatory marker levels were elevated in all cases, with CRP values ranging from 10 to 35 mg/dL and ESR values ranging from 12 mm/h to 65 mm/h, indicating significant systemic inflammation.

Despite intensive medical management, including the administration of inhaled nitric oxide and/or sildenafil in all four infants to address severe pulmonary hypertension, clinical deterioration progressed. All patients required vasoactive support, signifying cardiovascular compromise. The duration of hospitalization widely varied, from 15 to 60 days, reflecting prolonged critical illness with escalating respiratory and cardiac failure.

Ultimately, the cause of death in three infants was refractory respiratory failure, whereas one infant succumbed to combined respiratory and cardiac failure. Collectively, these cases illustrate the severe and rapidly progressive nature of pertussis in unvaccinated young infants and highlight the constellation of extreme leukocytosis, multisystem involvement, and refractory cardiopulmonary failure that characterizes fatal disease.

## 4 Discussion

This single-center retrospective study provides a detailed analysis of the clinical, laboratory, and therapeutic characteristics associated with death in critically ill infants diagnosed with critical *Bordetella pertussis* in the southwest region of Saudi Arabia. Our findings underscore the devastating effects of this vaccine-preventable disease, particularly in young, unimmunized infants aged < 3 months, which aligns with global observations regarding pertussis severity and mortality in this vulnerable population (20). The constellation of clinical features observed, namely, severe pneumonia, high fever, rapid respiratory distress culminating in respiratory failure, and frequent cardiac involvement, paints the picture of a rapidly progressive and overwhelming systemic inflammatory response.

### 4.1 Clinical presentation and demographics

The observed median age of 3 months at presentation aligns with established epidemiological data, indicating that young infants to have completed their primary pertussis vaccination series are at the highest risk of severe disease and mortality (21). This finding reinforces the importance of maternal vaccination strategies to passively protect newborns during that period of vulnerability (22). Simultaneously, the lack of a significant difference in age between survivors and non-survivors suggests that other factors, such as pre-existing comorbidities and the severity of clinical presentation, play more decisive roles in determining outcomes.

Our analysis revealed a strong association between specific clinical symptoms at presentation and increased case fatality rates. In particular, the significantly higher prevalence of apnea, hypoxemia, and seizures in non-survivors indicates rapid and profound respiratory and neurological compromise. Apnea, a well-recognized complication, is thought to result from paroxysmal coughing, airway inflammation, and involvement of the central nervous system. The observed hypoxemia likely reflects the effects of PTx on the pulmonary vasculature, which leads to ventilation–perfusion mismatch and impaired gas exchange (23). The presence of CHD may also be a significant predictor of mortality, as infants with CHD have reduced cardiorespiratory reserves, rendering them particularly susceptible to physiological stress.

### 4.2 Pathophysiology and laboratory findings

Fatal outcomes in these infants were strongly associated with laboratory abnormalities indicative of severe inflammation and multiorgan involvement. *B. pertussis* produces PTx, a key virulence factor that disrupts intracellular signaling pathways, ultimately leading to lymphocytosis and increased production of inflammatory cytokines (24). The markedly elevated WBC count and ALC observed in non-survivors reinforces the established role of hyperleukocytosis as a critical risk marker. These findings align with the literature, highlighting the mechanistic association among PTx-driven leukocytosis, increased blood viscosity, pulmonary microvascular obstruction, and subsequent pulmonary hypertension, leading to right-sided heart failure (25, 26).This association is supported by previous studies demonstrating that a WBC count > 50 × 10^9^/L significantly increases the risk of death, with a value > 100 × 10^9^/L acting as a strong predictor of mortality (21, 23, 27). In this cohort, hyperleukocytosis was more common among non-survivors compared with survivors; however, this difference did not reach statistical significance. This trend may suggest an association with disease severity, as elevated white blood cell counts have been linked to worse outcomes in severe pertussis. Nevertheless, the small number of non-survivors limits statistical power, and these findings should be interpreted with caution.

The significant elevation of the levels of CRP and other acute-phase proteins in non-survivors also indicates a more pronounced inflammatory cascade than that in survivors (23). Elevated levels of liver enzymes (AST and ALT) and decreased platelet counts indicate secondary hepatic injury and consumptive coagulopathy, highlighting the systemic, multiorgan nature of malignant pertussis (23, 26).

### 4.3 Therapeutic interventions and outcomes

Disease progression may continue despite intensive care interventions, highlighting the limitations of the current treatment strategies. Although macrolide therapy is recommended to eradicate nasopharyngeal carriage and limit transmission, its effectiveness in altering the clinical course beyond the prodromal phase remains highly limited (21, 23, 28). This limitation was evident in our cohort, where macrolide therapy was administered to 92.4% of survivors and 100% of non-survivors, without significantly preventing rapid clinical deterioration in fatal cases. However, the efficacy of macrolide therapy cannot be inferred from these observational data, and further well-designed prospective studies are required. Furthermore, because diagnoses were exclusively confirmed by PCR, Bordetella pertussis strains were not isolated via culture, precluding assessment of macrolide resistance in this study.

In our cohort, the utilization of aggressive interventions, including exchange transfusion, hydroxyurea, inhaled nitric oxide/sildenafil, and corticosteroids, was significantly higher in non-survivors than in survivors, reflecting a more refractory disease course.

Extreme hyperleukocytosis necessitates rapid leukoreduction strategies to mitigate pulmonary vascular resistance (21, 27). However, the association between exchange transfusion and death in our cohort suggests that such interventions are often implemented only after advanced cardiovascular failure or irreversible pulmonary damage occurs. Similarly, increased use of hydroxyurea to pharmacologically control hyperleukocytosis may be significantly slow (typically taking 5–7 days) to counteract the rapid progression of the disease in critically ill infants (29, 30). The relative ineffectiveness of potent pulmonary vasodilators (inhaled nitric oxide and sildenafil) further supports the hypothesis that pulmonary vascular obstruction in malignant pertussis is mechanical (owing to leukocyte clumps) rather than purely vasospastic; hence, vasodilator therapy alone is insufficient in the absence of effective early leukoreduction (23). The more frequent use of corticosteroids in non-survivors likely reflects a final attempt to mitigate severe systemic inflammation in patients with a poor prognosis (31).

### 4.4 Public health implications and limitations

The attribution of death to refractory respiratory failure and combined respiratory and cardiac failure emphasizes the importance of early diagnosis and prevention. The reasonably high case fatality proportion observed in this cohort further underscores the need to implement and reinforce maternal immunization strategies and timely infant vaccination programs in southwestern Saudi Arabia.

Our study is limited by its relatively small sample size, which constrains the generalizability of the findings. Future research should involve larger, multicenter cohorts to better define the clinical and laboratory characteristics of severe pertussis and to identify potential biomarkers for early diagnosis. Further investigation into the genetic diversity of circulating B. pertussis strains may also help clarify their contribution to virulence. In addition, evaluation of the efficacy and optimal timing of adjunctive interventions, including early exchange transfusion protocols, is needed to improve clinical outcomes. Another limitation is that screening for other respiratory pathogens was not performed, which may have hindered the assessment of coinfections or alternative etiologies, particularly among patients with a clinical suspicion of pertussis but negative diagnostic test results. Lastly, incomplete documentation of immunization status among survivors limited our ability to evaluate the association between vaccination and clinical outcomes.

## 5 Conclusion

This study highlights that critical pertussis mainly affects young, unimmunized infants aged < 3 months, with severe symptoms and reasonably high case fatality rate despite intensive treatment. Strengthening maternal vaccination, timely infant immunization, early risk identification, and prompt and aggressive interventions are crucial for improving outcomes. Further studies and public health efforts are required to reduce the disease burden in this vulnerable population.

## Data Availability

The raw data supporting the conclusions of this article will be made available by the authors, on request and without undue reservation.
